# Multiple management strategies to prolong gestational period after radical trachelectomy

**DOI:** 10.1002/ccr3.2400

**Published:** 2019-08-30

**Authors:** Masami Ito, Satoshi Yoneda, Arihiro Shiozaki, Kaori Fukuta, Noriko Yoneda, Shigeru Saito

**Affiliations:** ^1^ Department of Obstetrics and Gynecology University of Toyama Toyama Japan

**Keywords:** gauze compression, massive genital bleeding, multiple management strategies, pregnancy after trachelectomy, preterm premature rupture of membranes

## Abstract

Preterm premature rupture of membranes and massive genital bleeding in the second trimester are serious obstetrical problems in pregnancy after trachelectomy. We had managed a twin post‐trachelectomy pregnancy by multiple strategies, and two healthy infants were delivered at 32+5 weeks, although the optimum management for such patients is unknown.

## 1 INTRODUCTION

Radical trachelectomy (RT) is a fertility‐preserving surgical alternative to hysterectomy for early‐stage cervical cancer in patients of childbearing age. In RT, the cervix is removed and the uterine body is connected to the upper part of the vagina.^1^ However, post‐trachelectomy women are at high risk of preterm birth, because preterm premature rupture of membranes (pPROM) associated with ascending inflammation/infection in the vagina or cervix causes spontaneous preterm delivery (SPTD). It has been reported that the overall prematurity rate in singleton pregnancy after fertility‐sparing surgery (including RT) is 38%.^2^ Moreover, massive genital bleeding from the venous plexus of the residual cervix in the second trimester is regarded as a characteristic feature during pregnancy after RT, resulting in preterm birth.^3^ The preterm delivery rates in twin pregnancy after RT are higher than that of a singleton pregnancy after RT. Therefore, management strategies to prevent pPROM and for massive bleeding are necessary in pregnancy after trachelectomy. We would like to introduce the multiple management strategies employed for a dichorionic diamniotic (DD) twin pregnancy after RT, which concluded with the delivery of two healthy infants.

## 2 CASE REPORT

A 34‐year‐old (gravid 1, para 1) woman was diagnosed with a high‐grade squamous intraepithelial lesion by Pap smear and carcinoma in situ by punch biopsy. She underwent conisation using a loop electrosurgical excision procedure. Histological examination indicated microinvasive adenosquamous carcinoma, positive surgical margin with lymphovascular space invasion (LVSI). She was diagnosed with cervical cancer, FIGO stage IA1. However, magnetic resonance imaging (MRI) and computerized tomography (CT) showed no extended lesion of the cervical cancer. She and her husband wanted to preserve fertility and she underwent abdominal semiradical trachelectomy, also known as type II or modified radical trachelectomy at 35 years old (April 2013). The surgical procedures comprised bilateral complete pelvic lymphadenectomy. The obturator lymph node was sent for frozen section analysis and identified as histologically negative. Uterine arteries were preserved. Then vaginectomy was performed, and the cervix was excised approximately 1 cm below the internal os and examined by frozen section evaluation. The margins were secure, and permanent cerclage was placed abdominally using Ethibond polyester sutures (nonabsorbable suture). Finally, the uterus was reconstructed to the vagina. There were no residual tumors in the specimen and no metastasis in the pelvic lymph nodes. The pathological diagnosis was microinvasive adenosquamous carcinoma, pT1a1N0M0.

Six months later, all gynecological examinations, including Pap smears, MRI, and CT revealed the absence of recurrent or residual disease. Therefore, she was encouraged to become pregnant. Because she did not get pregnant naturally, she was referred to a fertility specialist at 36 years old. Her anti‐Müllerian hormone level was 3.89 ng/mL, and her husband's sperm had no problem, but bilateral hydrosalpinx of her fallopian tubes was found. Therefore, in vitro fertilization was performed. However, she did not achieve pregnancy after a total of six separate blastocyst transfers (Gardner's classification^4^ of transferred embryos: First time was 3AA; second, 4BA; third, 4BB in first oocyte retrieval. Fourth time was 4BB; fifth, 4BA; sixth, 5BA + 5BB in second oocyte retrieval). Although twin pregnancy after RT should be avoided, she and her husband strongly requested the transfer of two blastocysts after the third oocyte retrieval, and she became pregnant with DD twins. She was referred to our hospital again after confirmation of pregnancy, because she was at high risk of SPTD. We provided information on fetal reduction to the couple, but they wished to continue with both babies. Therefore, we proceeded to provide all possible support. Her cervical length at 10^+1^ weeks of gestation was 27 mm.

### 2.1 Treatment

**Figure 1:**
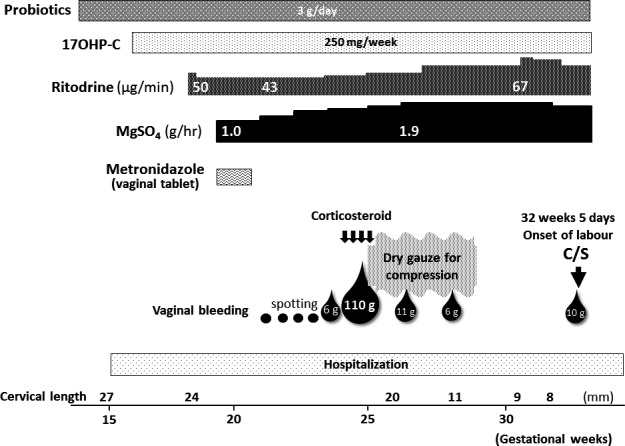
Clinical course and multiple strategies such as intake of oral probiotics, treatment to prevent inflammation/infection in the vagina or cervix, 17‐alpha‐hydroxyprogesterone caproate (17OHP‐C**)** administration, hospitalization, maintenance tocolysis, and using dry gauze for massive genital bleeding in the management of this dichorionic diamniotic twin pregnancy after trachelectomy are shown. At 32^+5^ wk, labor pains occurred, and she delivered two healthy infants (1806 g and 1705 g) by cesarean section. MgSO_4_, magnesium sulfate; cesarean section, C/S

The clinical course and multiple management strategies for this case are shown in Figure 1. To prevent pPROM, oral probiotics including *Clostridium butyricum*, *Streptococcus faecalis*, and *Bacillus mesentericus* (3 g/d) were prescribed^5^, ^6^, ^7^, ^8^ from 12 weeks of gestation. Metronidazole (vaginal tablet) was administered for bacterial vaginosis (BV) to prevent ascending inflammation and/or infection of the intra‐amnion.^9^ Additionally, 17‐alpha‐hydroxyprogesterone caproate (17OHP‐C)^10^, ^11^ was administered from 16 weeks. She was hospitalized for physical rest^9^ from 15^+1^ weeks. At 18 weeks of gestation, intravenous ritodrine, and at 20 weeks magnesium sulfate (MgSO_4_), were administered to control regular uterine contractions.^12^

**Figure 2:**
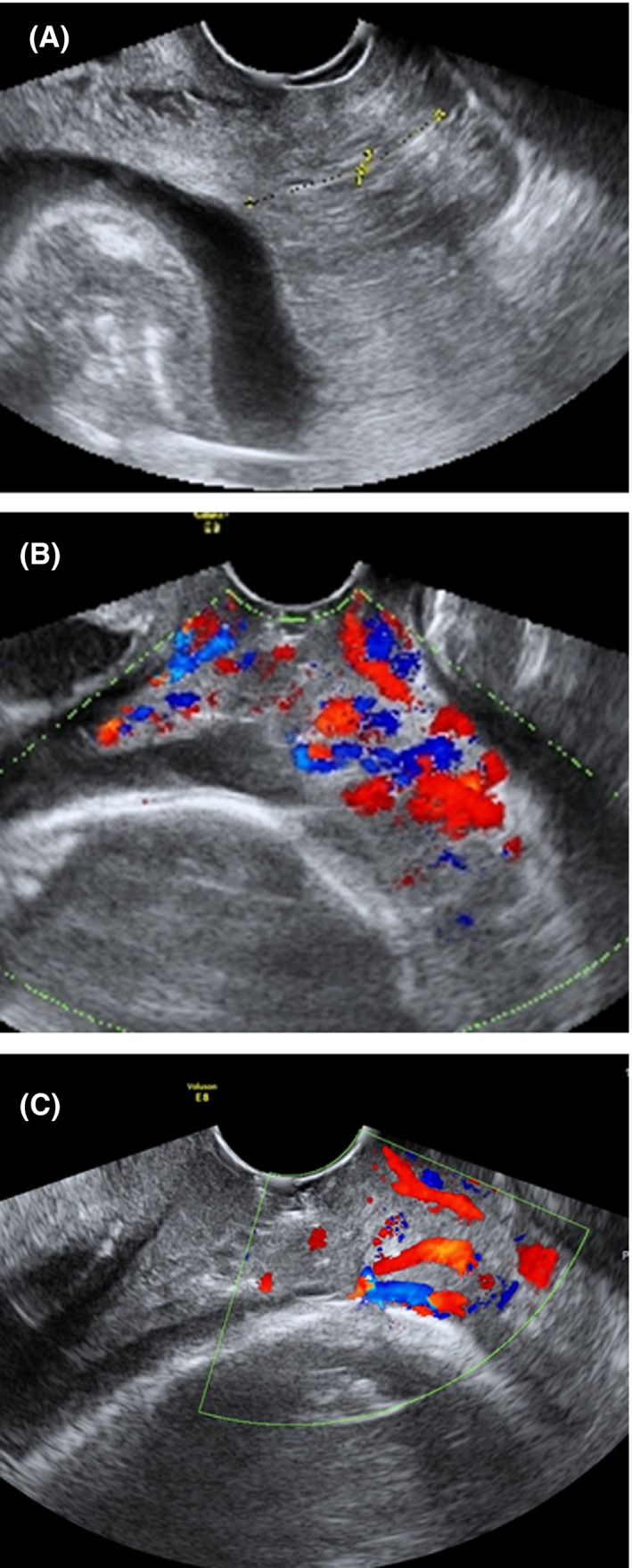
Serial measurements of the length of the ‘neo‐cervix’ were performed, and revealed that it had shortened during pregnancy. A, neo‐cervical length was 27 mm at 15^+1^ wk on admission. B, neo‐cervical length was 20 mm at 25^+6^ wk, and transvaginal ultrasonography showed abundant vascularity (red and blue) in the “neo‐cervix” region. C, neo‐cervical length was 8 mm at 31^+5^ wk and transvaginal ultrasonography showed abundant vascularity in the “neo‐cervix” region

Serial measurements of the length of the “neo‐cervix” were performed at least once a week during pregnancy. Vaginal ultrasonography showed increased vascularity in the “neo‐cervical” region (Figure 2), and slight genital bleeding occurred from 21 weeks of gestation. Because the frequency of uterine contractions and genital bleeding increased, corticosteroid for fetal lung maturation was administered (dexamethasone at 6 mg every 12 hours, a total of four times^13^) at 24^+5^‐24^+6^ weeks.

At 25^+6^ weeks, massive genital bleeding occurred from the neo‐cervix, and emergency cesarean section (C/S) was considered, when the bleeding failed to stop. However, fetal well‐being was good and their growth was normal (806 g, −0.4 SD and 803 g, −0.4 SD). Considering abnormal blood flow due to trachelectomy, we decided on conservative management and immediately tried to stop the bleeding by putting dry gauze for compression into the vagina. The dry gauze was replaced daily, and bleeding reduced gradually and stopped at 29 weeks.

### 2.2 Outcome and follow‐up

**Figure 3:**
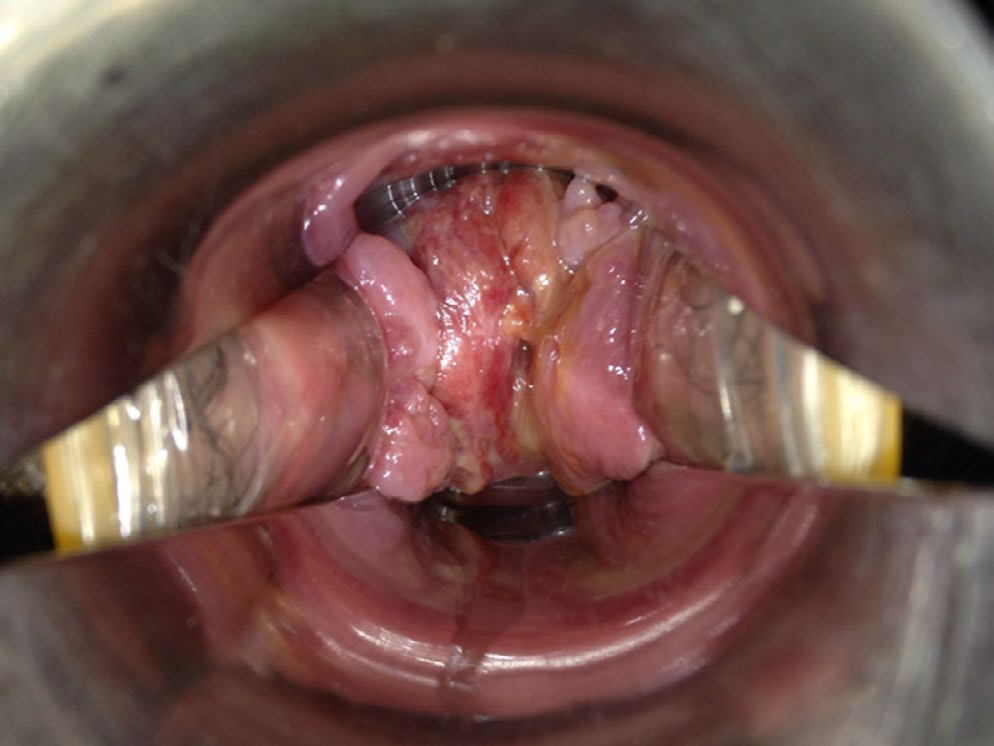
Neo‐cervix by vaginal speculum. We found no varix formation or abnormal blood vessels in neo‐cervix at 32^+5^ wk of gestation

Serial measurements of the length of the “neo‐cervix” were performed and revealed that it had shortened during pregnancy; 27 mm at 15^+1^ weeks on admission, 20 mm at 25^+6^ weeks, and 8 mm at 31^+5^ weeks (Figure 2). Because vaginal ultrasonography showed increased vascularity in the “neo‐cervical” region (Figure 2), massive genital bleeding might recur. However, we found no varix formation or abnormal blood vessels in the neo‐cervix using a vaginal speculum (Figure 3).

When MgSO_4_ was administered to control regular uterine contractions at 20 weeks, she only felt fatigue, but she had no serious side effects. Her renal function was good during pregnancy, and eGFR was 110‐150 (mL/min/1.73 m^2^). Electronic fetal monitoring (EFM) at 22 weeks revealed reassuring fetal status when the MgSO_4_ dose was 1.2 g/h and her serum Mg concentration was 4.9 mg/dL. Slight genital bleeding occurred from 21 weeks of gestation, but her hemoglobin (Hb) level stayed low around 7‐9 g/dL until delivery by administration of iron (Hb level: before pregnancy was 11.7 g/dL, 11.2 g/dL at 8 weeks, and 9.4 g/mL at 16 weeks). Blood transfusion was not given. Her blood pressure of 10 weeks was 102/65 mm Hg and stayed normal thereafter. Proteinuria was not detected during pregnancy.

At 32^+5^ weeks of gestation, labor pains occurred, and C/S was carried out immediately. Two preterm infants, weighting 1806 g (−0.3 SD) and 1705 g (−0.6 SD), were delivered and admitted to the neonatal intensive care unit. Although both infants were diagnosed with respiratory distress syndrome and treated immediately with surfactant using the INtubation‐SURfactant‐Extubation (INSURE) method,^14^ they were on room air at postnatal day 9 and later. They did not develop chronic lung disease (CLD), had no other major complications, and were discharged at 2770 g and 2444 g, respectively. The mother was discharged on postoperative day 6 without any complications.

Histological chorioamnionitis (h‐CAM) was not detected in first infant, but stage 1 h‐CAM was diagnosed in the second infant. Funisitis was not detected in either infant.

## 3 DISCUSSION

Only five reports on post‐trachelectomy “twin” deliveries (including six cases) have been published to date,^15^, ^16^, ^17^, ^18^, ^19^ using Pub‐Med and MEDLINE published in the English language between 1985 and 2018 with the key search word: “twin” and “trachelectomy”. These six cases are summarized in Table 1; the mean gestational age at birth was 29 weeks. The mean gestational age at birth in post‐trachelectomy “singleton” pregnancy was 33 weeks.^3^ Therefore, the gestational period in twin pregnancy was 4 weeks shorter than that in singleton pregnancies. Additional careful clinical management is necessary for “twin” pregnancy after trachelectomy.

**Table 1:** Summary of case reports of twin pregnancy after trachelectomy

Year	Stage/pathology	Conception	Chorionic	Gestational age (wk)/mode of delivery	Event and management	Birth weight (g)
2003^15^	Unknown/unknown	Unknown	Unknown	24/unknown	Unknown	Unknown
2003^16^	Unknown/unknown	IUI	Unknown	24/CS	24 wk pPROM, CAM	Unknown
	Unknown/unknown	IUI	MD	26/unknown	TTTS, HELLP syndrome	Unknown
2007^17^	IA1/adenocarcinoma	IVF (donor egg)	Unknown	30/emergency CS	Massive vaginal bleeding at 30 wk.	1410
					Placenta accreta → hysterectomy	1510
2009^18^	IA2/SCC	IVF	Unknown	36/unknown	Unknown	Unknown
2014^19^	IB1/SCC	Spontaneous	MD	34/elective CS	Unknown	2360 1720
This case	IA1/adenosquamous	IVF	DD	32/emergency CS	Multiple strategies to prevent pPROM.	1806
					Dry gauze for massive genital bleeding at 25 wk.	1705

Abbreviations: CAM, chorioamnionitis; CS, cesarean section; DD, Dichorionic diamniotic; IUI, Intrauterine insemination; IVF, in vitro fertilization; MD, Monochorionic diamniotic; pPROM, preterm premature rupture of membranes; SCC, squamous cell carcinoma; TTTS, twin‐to‐twin transfusion syndrome.

In this DD twin case, multiple management strategies that might be effective to prevent pPROM were administered. Intake of probiotic food is associated with a reduced risk of SPTD,^8^, ^20^ so we prescribed oral probiotics (3 g/d) including *Clostridium butyricum*, *Streptococcus faecalis,* and *Bacillus mesentericus*. In particular, *Clostridium* is associated with regulatory T cells,^6^ which are necessary to maintain pregnancy. We reported that *Clostridium* subcluster XVIII and *Clostridium* subcluster XIVa were significantly lower in the intestinal microbiota in SPTD cases.^5^ Additionally, oral probiotics including *Clostridium* for pregnancy with a history of SPTD have been reported to reduce SPTD.^7^

Although treatment for BV was not able to reduce SPTD significantly,^21^ it is recommended for pregnancy after trachelectomy.^22^, ^23^ Therefore, metronidazole (vaginal tablet) was administered for BV, considering the risk of an ascending inflammation/infection.

Administration of progesterone agents could prevent SPTD,^10^ but in multiple pregnancies, it does not appear to be associated with a reduction in the risk of preterm birth.^24^ Thus, it is not known whether 17OHP‐C is effective for preventing preterm labor (PTL). However, 17OHP‐C, which might be effective in limited PTL cases with mild sterile intra‐amniotic inflammation,^11^ was administered prophylactically from 16 weeks.

She was hospitalized for physical rest from 15^+1^ weeks. Hospitalization as well as progesterone is also recommended in a standardized protocol for pregnant women after radical RT.^9^ Additionally, intravenous ritodrine and MgSO_4_ were administered for regular uterine contractions during hospitalization. Maintenance tocolysis might also be effective for PTL cases with mild intra‐amniotic inflammation (in this case, stage 1 h‐CAM was diagnosed in the second infant), lean women (her prepregnancy body mass index was 18.9), and cerclage (she underwent transabdominal cervical cerclage).^12^

On the other hand, massive genital bleeding occurred from the neo‐cervix at 25^+6^ weeks of gestation, and emergency C/S was considered. However, the abnormal bleeding decreased gradually by putting dry gauze for compression in the vagina daily. Only one report has mentioned that vaginal tamponade with gauze for massive bleeding might be effective.^3^

Although there is no guideline for optimal management for pregnant women after RT at present, a post‐trachelectomy twin pregnancy was concluded successfully using these various management strategies, and two healthy infants were delivered. These practical multidisciplinary strategies to prevent pPROM and for massive bleeding in the second trimester may be considered to be useful in the management of pregnancy after trachelectomy.

Lastly, we discuss the initial surgical management of this case. She was diagnosed with cervical cancer, FIGO stage IA1, but with a positive surgical margin with LVSI. She underwent abdominal‐modified RT in April 2013. Recently, however, there has been a trend toward more conservative surgery, and good oncologic and obstetric outcomes of simple trachelectomy or cone.^25^ We considered that repeat cone biopsy and sentinel lymph node mapping may be a better alternative for such a patient at the present time. We hope that these less invasive procedures for patients with early‐stage disease will reduce the prematurity rate in pregnancy after fertility‐sparing surgery.

## CONFLICT OF INTEREST

None declared.

## AUTHOR CONTRIBUTIONS

MI: involved in the conception, manuscript writing, acquisition of data and editing. SY: drafted the manuscript, edited for scientific merit, and approved final draft. AS: drafted the manuscript. KF: involved in patient management. NY: involved in patient management. SS: contributed to revisions and final approval.
